# The Texas Dental Association

**Published:** 1900-03

**Authors:** J. W. David, J. G. Fife

**Affiliations:** President; Dallas, Texas; Secretary; Dallas, Texas


### The Texas Dental Association.

The next annual meeting of the Texas Dental Association will meet in Dallas, May 15th, 16th, and 17th. Reduced railroad rates have been secured within the State, and a large attendance is expected. The profession cordially invited.

J. W. DAVID,

President.

J. G. FIFE, Secretary.

Dallas, Texas.

